# Functional characterization of Griscelli syndrome type 2 sine albinism in Japanese patients

**DOI:** 10.70962/jhi.20250270

**Published:** 2026-05-07

**Authors:** Tatsuhiko Tanaka, Akira Sugawara, Ryuhei Yasuoka, Kimiyoshi Sakaguchi, Osamu Natsume, Kentaro Haga, Yuto Maruta, Akie Kobayashi, Tomohiko Sato, Erina Saito, Satoko Minakawa, Yuiko Hirata, Hirofumi Shibata, Takahiro Yasumi, Masaki Shimizu, Hirokazu Kanegane, Ko Kudo, Mitsunori Fukuda, Kiminori Teui

**Affiliations:** 1 Department of Pediatrics, Hirosaki University Graduate School of Medicine, Hirosaki, Japan; 2 Laboratory of Membrane Trafficking Mechanisms, Department of Integrative Life Sciences, Graduate School of Life Sciences, Tohoku University, Sendai, Japan; 3 Department of Pediatrics, Hamamatsu University School of Medicine, Shizuoka, Japan; 4 Department of Neuroanatomy, Cell Biology and Histology, Hirosaki University Graduate School of Medicine, Hirosaki, Japan; 5 Department of Dermatology, Hirosaki University Graduate School of Medicine, Hirosaki, Japan; 6 Department of Pediatrics, Graduate School of Medicine, Kyoto University, Kyoto, Japan; 7 Department of Pediatrics, Perinatal and Maternal Medicine, Graduate School of Medical and Dental Sciences, Institute of Science Tokyo, Tokyo, Japan; 8 Department of Child Health and Development, Graduate School of Medical and Dental Sciences, Institute of Science Tokyo, Tokyo, Japan

## Abstract

Griscelli syndrome type 2 (GS2), caused by biallelic *RAB27A* variants, is classically associated with hypopigmentation and life-threatening hemophagocytic lymphohistiocytosis (HLH). However, a few patients exhibit normal pigmentation, termed GS2 sine albinism. We identified two unrelated Japanese patients with this presentation caused by compound heterozygous *RAB27A* variants, representing the first functionally characterized cases among Japanese patients. Functional studies were performed using *RAB27A*-null cell systems to evaluate the two allele pairs: p.Trp73Arg/p.Val143Ala (patient 1) and p.Ser106PhefsTer18/p.Ser115Arg (patient 2). The truncating allele p.Ser106PhefsTer18 and the missense allele p.Trp73Arg failed to restore peripheral melanosome distribution and lost binding to both melanophilin (MLPH) and MUNC13-4. In contrast, p.Val143Ala and p.Ser115Arg preserved MLPH interaction but showed reduced binding to MUNC13-4. These findings demonstrate that *RAB27A* variants can dissociate melanosome transport from immune effector function in an allele-dependent manner. Importantly, the absence of hypopigmentation does not exclude GS2. Combined genetic and functional analyses are essential for precise variant interpretation and for guiding timely curative intervention in GS2.

## Teaser

Griscelli syndrome type 2 typically presents with hypopigmentation and HLH, but some patients retain normal pigmentation. Tanaka et al. functionally characterize compound heterozygous *RAB27A* variants in Japanese patients, showing allele-specific dissociation of melanosome transport and cytotoxic function, underscoring the need for integrated genetic and functional diagnosis.

## Introduction

Griscelli syndrome type 2 (GS2) is caused by biallelic variants in *RAB27A* and is classically characterized by hypopigmentation occurring with hemophagocytic lymphohistiocytosis (HLH) (1, 2, 3). *RAB27A* encodes a small GTPase that tethers melanosomes to the actin-based motor machinery; in melanocytes, it engages melanophilin (MLPH; also known as SLAC2-A) and myosin Va to drive actin-based melanosome transport at the cell periphery (4, 5, 6, 7, 8), whereas in cytotoxic lymphocytes, it engages MUNC13-4 to prime and release lytic granules (9, 10, 11). Over the past decade, “sine albinism” phenotypes have been recognized where pigmentation is preserved despite severe immune dysregulation (12, 13, 14, 15). “Sine albinism” is used here to denote the absence of clinically appreciable cutaneous and ocular hypopigmentation. Mechanistically, allele- and tissue-specific effects appear central: preservation of MLPH engagement can maintain melanosome transport, while impaired interaction with MUNC13-4 disrupts cytotoxic granule release and is associated with severe, sometimes refractory, HLH (12, 13, 14, 15).

Reports of GS2 from East Asia are limited, and sine albinism may be underrecognized when pigmentation is preserved (16, 17). Here, we report two unrelated children with GS2 sine albinism who carried compound heterozygous *RAB27A* variants. Both exhibited normal pigmentation and profound degranulation defects. By integrating clinical phenotyping with genetic testing and functional analyses in cultured RAB27A-deficient melanocytes together with effector-binding assays, we depict how specific allele combinations can preserve melanocyte transport yet compromise cytotoxic exocytosis, providing the first functionally validated Japanese cases of GS2 with sine albinism.

## Results

### Clinical features and genetic findings

This study included two unrelated Japanese patients who presented with hyperinflammatory disease consistent with GS2 but exhibited normal pigmentation.

### Patient 1

**Figure 1.**
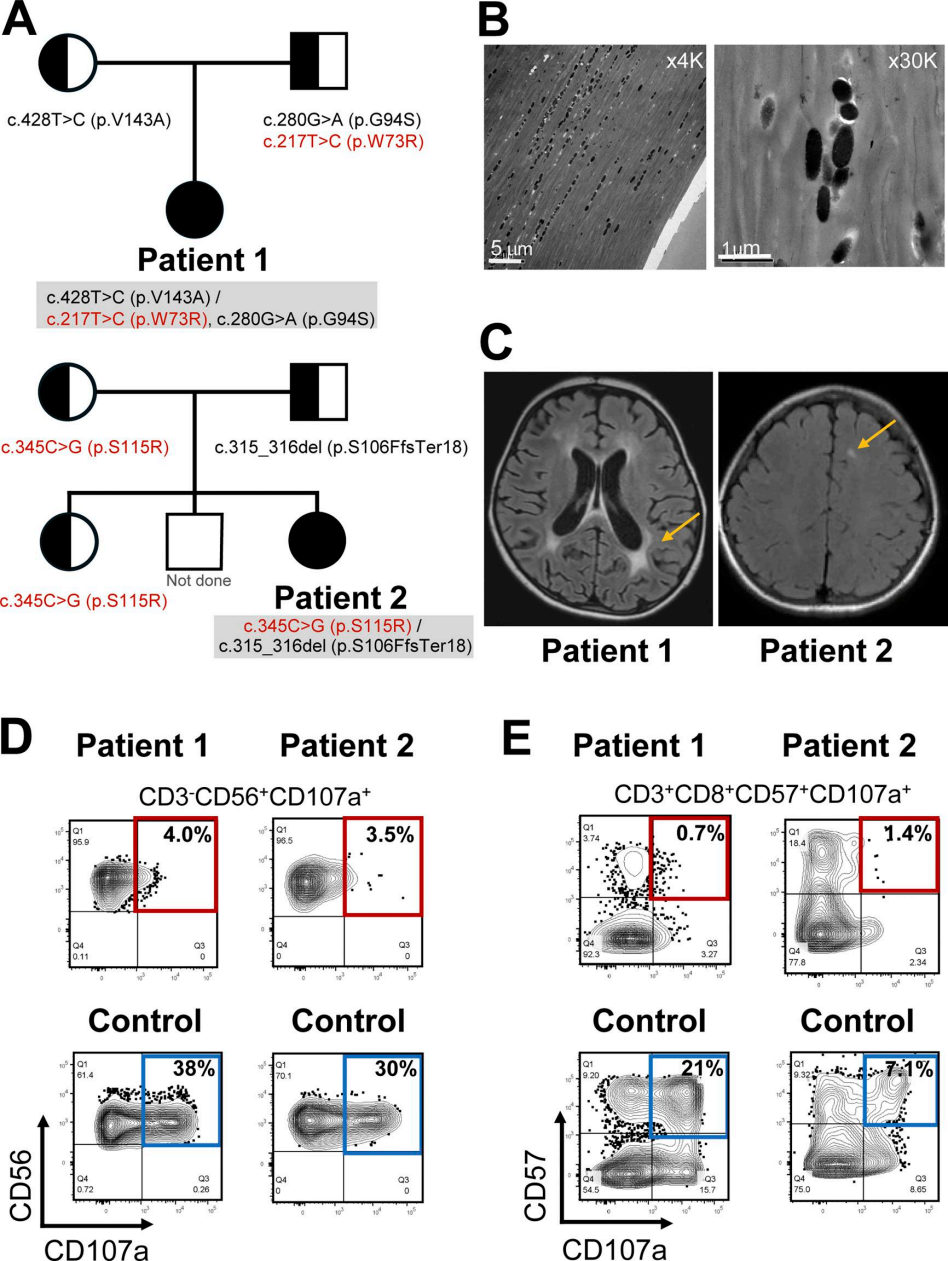
**Clinical, radiological, and immunological findings in two patients with GS2. (A)** Pedigrees and results of *RAB27A* sequencing. Patients 1 and 2 carried compound-heterozygous combinations of one previously reported allele and one novel allele. Novel variants are highlighted in red. **(B)** Transmission electron microscopy of a hair shaft from patient 1 showing normal, evenly distributed mature melanosomes along the hair-shaft cortex (left, ×4,000; right, ×30,000), consistent with normal pigmentation. **(C)** Axial T2 FLAIR brain MRI from both patients demonstrating white-matter lesions (yellow arrows) compatible with CNS-HLH. **(D)** Natural killer (NK) cell degranulation assay. NK cells were defined as CD3^−^CD56^+^ cells, and degranulation was quantified as the percentage of CD107a^+^ cells within this population. Degranulation was markedly reduced in patients 1 (4.0%) and 2 (3.5%) compared with healthy controls (30–38%). **(E)** CD8^+^ T cell degranulation assay under P815+OKT3 stimulation. CD8^+^ T cells were defined as CD3^+^CD8^+^ cells, and degranulation was quantified as the percentage of CD107a^+^ cells (CD57^+^ subset) after IL-2 treatment. Responses were severely impaired in patients 1 (0.7%) and 2 (1.4%) compared with controls (17–21%) after 2-h stimulation. Healthy control donors were not age-matched. Due to limitations in sample availability and clinical constraints, the assay was performed once per patient, and the flow cytometry plots shown correspond to the data obtained for each individual. These findings demonstrate profound defects in NK and cytotoxic T cell degranulation despite normal pigmentation, consistent with *RAB27A*-related GS2 sine albinism.

A previously healthy 3-year-old girl presented with a 10-day history of fever, tachypnea, generalized edema, and hepatosplenomegaly. Laboratory findings showed pancytopenia (white blood count [WBC] 2.33 × 10^9^/liter; hemoglobin 8.8 g/dl; platelets 42 × 10^9^/liter), hypertriglyceridemia (327 mg/dl), hypofibrinogenemia (56 mg/dl), and markedly elevated ferritin (1,897 ng/ml) and soluble IL-2 receptor (sIL-2R, 23,202 U/ml). Bone marrow examination indicated hemophagocytosis. Neurologic examination revealed bilateral ankle dorsiflexion restriction, hyperreflexia, and ankle clonus, resulting in loss of ambulation. No cutaneous hypopigmentation, silvery hair, or iris hypopigmentation was observed. Targeted next-generation sequencing identified three *RAB27A* missense variants—p.Val143Ala (c.428T>C), p.Gly94Ser (c.280G>A) (rare SNP previously reported in the database), and the novel p.Trp73Arg (c.217T>C). Parental testing confirmed compound heterozygosity, with p.Val143Ala inherited maternally and p.Trp73Arg paternally (Fig. 1 A, upper panel).

Hair-shaft transmission electron microscopy (TEM) showed preserved, evenly distributed mature melanosomes (Fig. 1 B), consistent with intact melanosome transport despite immune dysfunction. Brain magnetic resonance imaging (MRI) and cerebrospinal fluid studies demonstrated abnormalities compatible with central nervous system-HLH (CNS-HLH) (Fig. 1 C, left panel). These findings were consistent with CNS involvement in HLH, rather than a primary neurodegenerative process. Flow cytometric CD107a degranulation assays revealed markedly reduced NK and CD8^+^ T cell degranulation (Fig. 1, D and E).

The patient received HLH-2004 induction therapy (dexamethasone, etoposide, and cyclosporine), achieving initial remission. Following a CNS relapse, she underwent re-induction with dexamethasone, ruxolitinib (10 mg/day), and four weekly intrathecal injections of methotrexate plus hydrocortisone and subsequently received unrelated umbilical cord blood transplantation with reduced-intensity conditioning. Neutrophil engraftment occurred on day 21, and she remains relapse-free without graft-versus-host disease or neurologic sequelae 1 year after transplant.

### Patient 2

An 11-year-old girl experienced recurrent HLH-like hyperinflammatory episodes over 2 mo, presenting with fever, tonsillar swelling, cervical lymphadenopathy, hepatosplenomegaly, pancytopenia, hyperferritinemia, and elevated sIL-2R. No hypopigmentation or silvery hair was noted, and ophthalmologic examination revealed normal iris pigmentation. Targeted sequencing identified compound-heterozygous *RAB27A* variants: a missense p.Ser115Arg (c.345C>G) and a paternal frameshift p.Ser106PhefsTer18 (c.315_316del), confirmed to be in trans (Fig. 1 A, lower panel).

During the fourth episode, she developed new-onset left hemiparesis. Brain MRI showed an abnormal T2 white-matter signal consistent with CNS involvement in the context of new-onset focal neurological deficits (Fig. 1 C, right panel). Laboratory evaluation revealed pancytopenia (WBC 1.4 × 10^9^/liter; hemoglobin 9.2 g/dl; platelets 15 × 10^9^/liter), ferritin 1,222 ng/ml, sIL-2R 8,821 U/ml, and markedly elevated cytokines (IL-18 10,950 pg/ml; CXCL9 12,753 pg/ml; sTNFR2 44,364 pg/ml). CD107a degranulation assays revealed severely impaired NK and CD8^+^ T cell degranulation (Fig. 1, D and E).

The first four inflammatory episodes resolved without immunosuppressive therapy. The fifth episode required HLH-2004-based induction therapy, which achieved only a transient remission. The disease relapsed early, and subsequent treatment with modified cyclophosphamide, doxorubicin, vincristine, and prednisone (CHOP) therapy and methylprednisolone pulse therapy failed to achieve adequate disease control. The patient subsequently underwent myeloablative conditioning followed by unrelated umbilical cord blood transplantation. However, HLH remained refractory, and the patient died on posttransplant day 18.

### Ultrastructural analysis

Hair-shaft TEM in patient 1 showed evenly distributed, mature melanosomes within the hair cortex (Fig. 1 B). These findings are consistent with preserved melanosome transport, likely due to retained MLPH interaction in the p.Trp73Arg and p.Val143Ala variant combination.

### Functional degranulation assays

To assess cytotoxic function, we performed flow cytometric CD107a degranulation assays. NK cell degranulation was markedly reduced in both patients (4.0% and 3.5%) compared with healthy controls (30–38%) (Fig. 1 D). Similarly, CD8^+^ T cell degranulation after 48-h stimulation was severely impaired (0.7% and 1.4% vs. 17–21% in controls) (Fig. 1 E). These findings demonstrate a consistent defect in degranulation across both cytotoxic lymphocyte subsets.

### Functional analysis of RAB27A carrying a novel Trp73Arg variant on melanosome transport in melanocytes in patient 1

**Figure 2.**
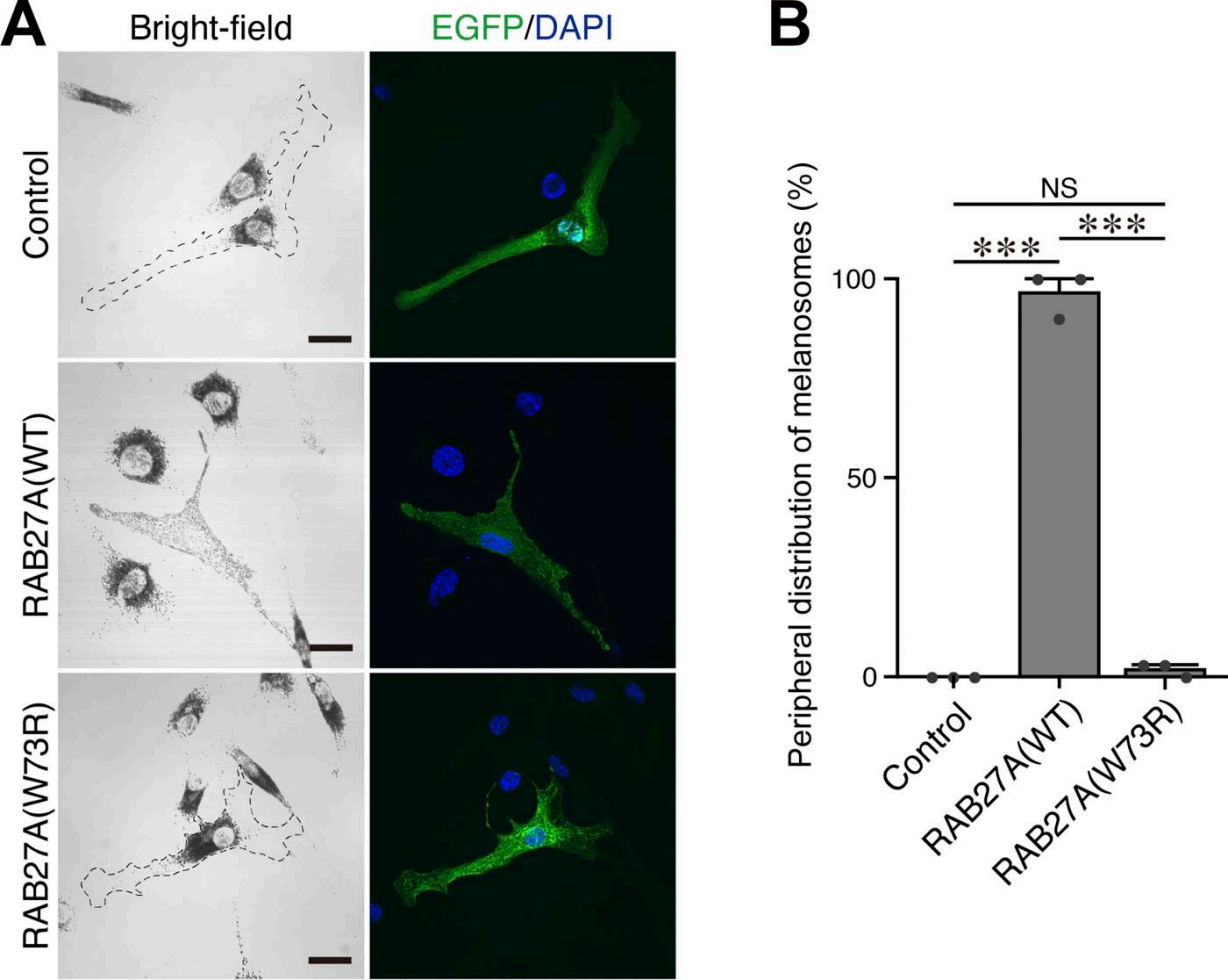
**RAB27A(Trp73Arg) did not restore peripheral melanosome distribution in RAB27A-deficient melanocytes. (A)** Typical images of melan-ash cells transiently expressing EGFP alone (control; top panels), EGFP-tagged RAB27A(WT) (middle panels), and EGFP-tagged RAB27A(Trp73Arg) (indicated as W73R; bottom panels). Cells with perinuclear aggregation were outlined with broken lines. Scale bars, 20 μm. **(B)** The percentages of cells showing peripheral melanosome dispersion in A. The error bars represent the means ± SE of data obtained in three independent experiments (*n* = 30 cells in each experiment), which were performed on different days. ***P < 0.001; NS, not significant (one-way ANOVA and Tukey’s test).

To investigate the impact of the Trp73Arg variant in RAB27A-mediated melanosome transport in melanocytes, we transiently expressed enhanced green fluorescent protein (EGFP)–tagged RAB27A(Trp73Arg) in melan-ash cells (an immortal mouse melanocyte cell line [18]), which genetically lack *RAB27A*, resulting in a typical melanosome aggregation phenotype around the nucleus (Fig. 2 A, top left panel). Re-expression of EGFP-RAB27A(wild type [WT]) in melan-ash cells completely rescued the RAB27A-deficient phenotype (Fig. 2 A, middle panels), and >90% of the cells exhibited peripheral melanosome distribution (i.e., normal phenotype in WT cells) (Fig. 2 B). In contrast, expression of EGFP-RAB27A(Trp73Arg) failed to restore peripheral melanosome distribution, similar to EGFP expression alone (Fig. 2 A, bottom panels; Fig. 2 B). These results indicated that RAB27A(Trp73Arg) was incapable of mediating transport of melanosomes to the cell periphery in cultured melanocytes.

### Effect of the Trp73Arg variant of RAB27A on binding activity toward MLPH and MUNC13-4

**Figure 3.**
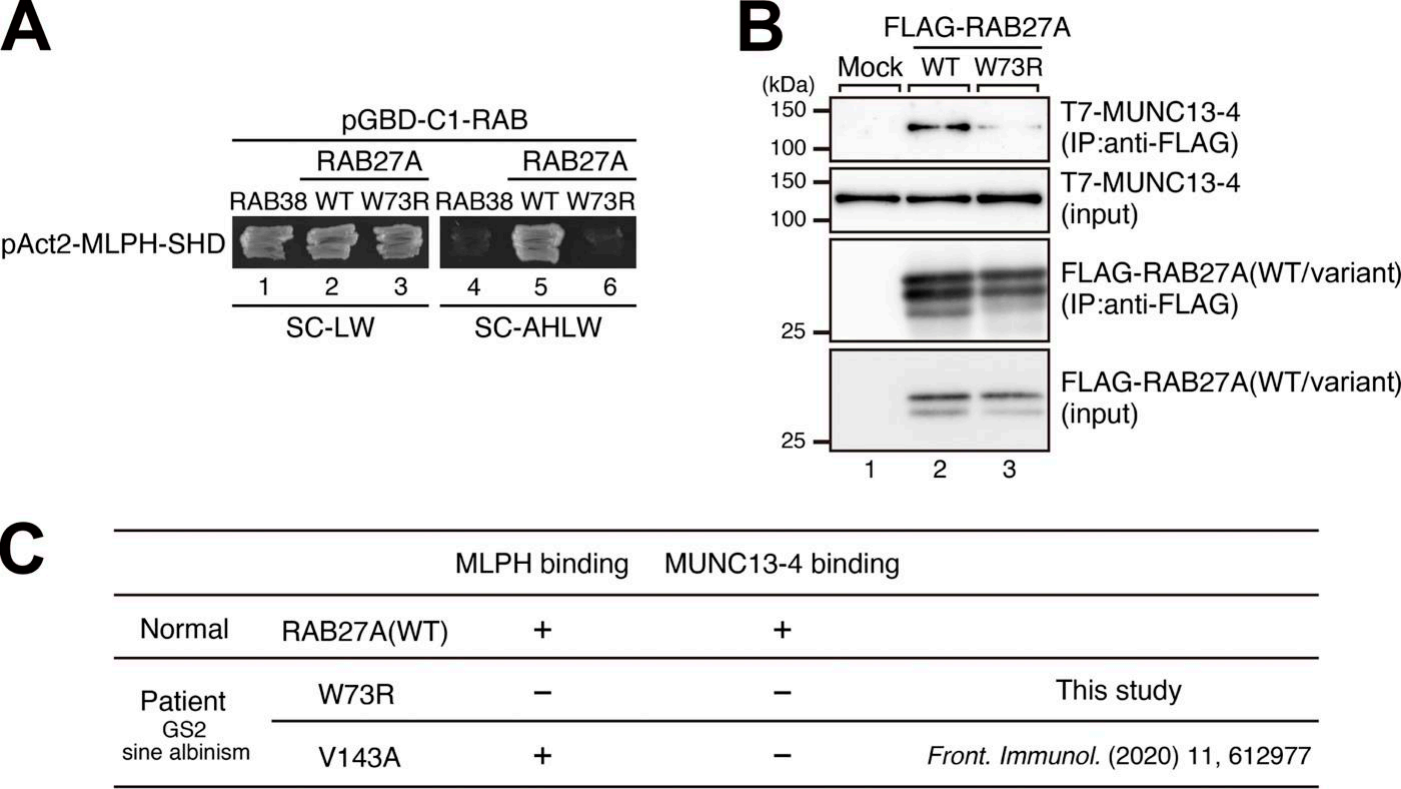
**RAB27A(Trp73Arg) was unable to bind to RAB effectors that function in melanocytes (MLPH) and CTLs (MUNC13-4). (A)** Interactions of RAB27A (WT or Trp73Arg; indicated as W73R) with MLPH-SHD as assessed using yeast two-hybrid assays. RAB38, another melanosomal protein that does not bind to MLPH, was used as a negative control. **(B)** Interactions of FLAG-RAB27A (WT or Trp73Arg) with T7-MUNC13-4 as assessed by co-immunoprecipitation assays in COS-7 cells. Co-immunoprecipitated T7-MUNC13-4 and immunoprecipitated (IP) FLAG-RAB27A were detected by immunoblotting with the antibodies indicated. The positions of the molecular mass markers (kDa) are shown on the left. **(C)** Summary of the binding activities of RAB27A (WT, Trp73Arg, and Val143Ala; indicated as V143A) toward MLPH, which functions in melanocytes, and MUNC13-4, which functions in CTLs. Data for RAB27A(Val143Ala) were obtained previously (14). Data in A and B are representative of the data obtained in two and three independent experiments, respectively, and similar results were obtained in each experiment. Source data are available for this figure: SourceData F3.

RAB27A is abundantly expressed in melanocytes and CD8^+^ cytotoxic T lymphocytes (CTLs), and the functional loss of RAB27A in these cells is known to cause GS2, which is characterized by hypopigmentation and immunodeficiency (8). Since RAB27A functions together with its cell type-specific effectors (7), e.g., MLPH in melanocytes (4, 5, 6) and MUNC13-4 in CTLs (9, 10, 11), we next investigated the effect of the Trp73Arg variant on the effector-binding ability of RAB27A. The results of yeast two-hybrid assays showed that MLPH did not recognize RAB27A(Trp73Arg) (Fig. 3 A, compare lanes 5 and 6), consistent with the fact that RAB27A(Trp73Arg) is unable to support actin-based melanosome transport in melan-ash cells (Fig. 2). Additionally, RAB27A(Trp73Arg) also showed markedly reduced binding ability toward MUNC13-4 in co-immunoprecipitation assays (Fig. 3 B, lane 3 in the top panel). Taken together, these results indicated that the Trp73Arg variant of RAB27A is likely to be a loss-of-function variant.

### Molecular diagnosis of patient 1 with heterozygous RAB27A(Trp73Arg/Val143Ala) variants

As summarized in the table of Fig. 3 C, the results of the binding activities of RAB27A(WT, Trp73Arg, and Val143Ala) toward MLPH and MUNC13-4 enabled us to understand the relationship between heterozygous *RAB27A(Trp73Arg/Val143Ala)* variants and GS2 sine albinism phenotypes. Both RAB27A(Trp73Arg) and RAB27A(Val143Ala) clearly showed the decreased MUNC13-4-binding activity (Fig. 3 B and [14]), reflecting HLH symptoms. RAB27A(Trp73Arg) was also unable to interact with MLPH and to recover the peripheral distribution of melanosomes in melan-ash cells (Fig. 2 and Fig. 3 A). In contrast, RAB27A(Val143Ala) had a normal MLPH-binding ability and mostly restored peripheral melanosome distribution in melan-ash cells (see [14] for details). Additionally, homozygous *RAB27A(Val143Ala)* variant did not display a hypopigmentation disorder (14). Thus, in melanocytes from the patient with heterozygous *RAB27A(Trp73Arg/Val143Ala)* variants, RAB27A(Val143Ala) is able to support actin-based melanosome transport and peripheral melanosome distribution, even though RAB27A(Trp73Arg) is completely nonfunctional, resulting in no albinism in this patient.

### Effect of novel *RAB27A* variants on melanosome transport in melanocytes in patient 2

**Figure 4.**
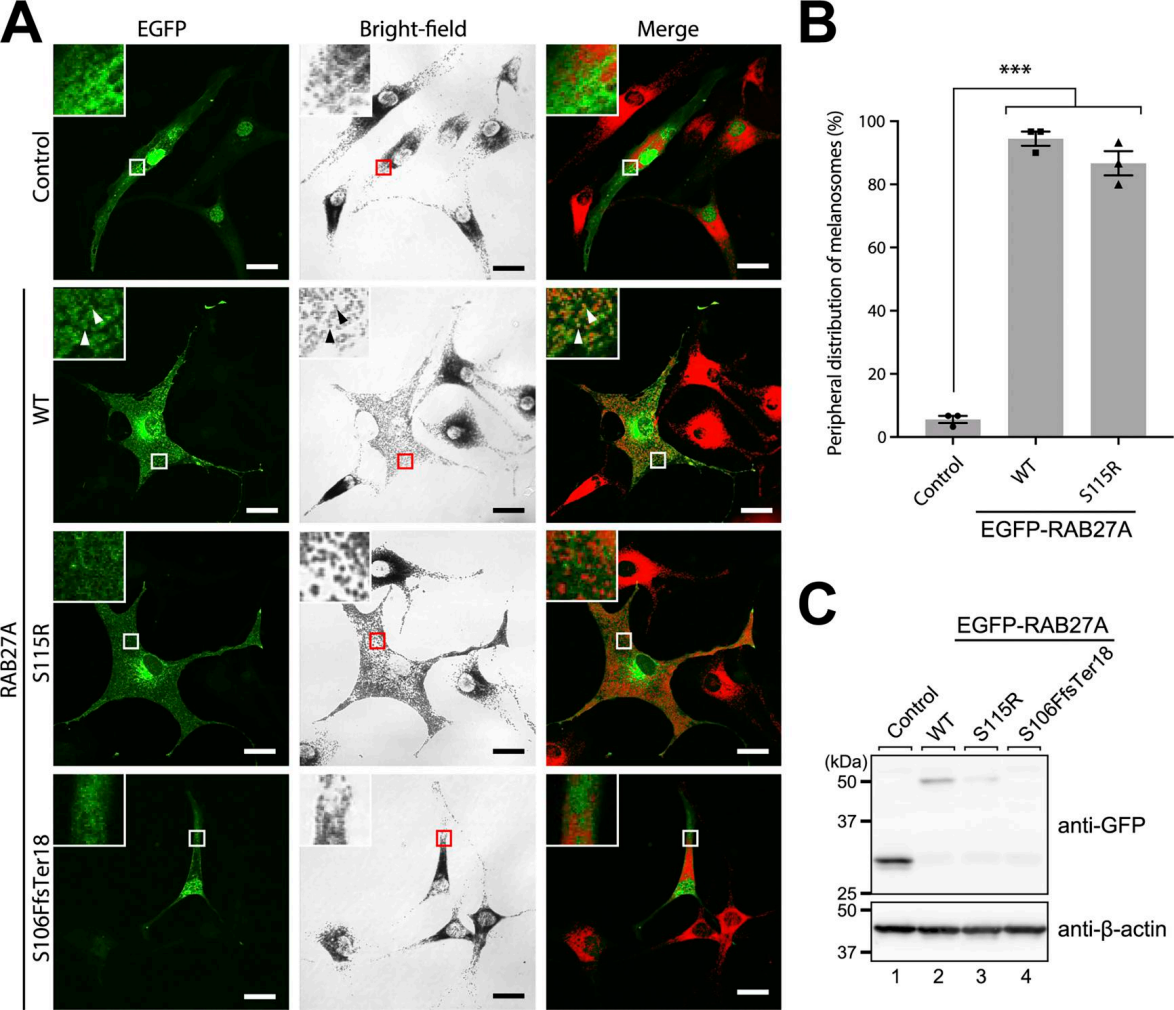
**Subcellular localization of melanosomes in RAΒ27A-deficient melanocytes transiently expressing RAΒ27A variants. (A)** EGFP alone (control) or EGFP-tagged RAB27A (WT, Ser115Arg [indicated as S115R], and Ser106PhefsTer18 [indicated as S106FfsTer18]) were transiently expressed in melan-ash cells. EGFP is shown in green, and melanosomes in the right panels (Merge) are pseudo-colored in red. The insets show magnified views of the boxed areas. The arrowheads indicate the colocalization between EGFP-RAΒ27A (WT) and melanosomes. Scale bars, 20 μm. **(B)** Percentage of cells showing peripheral melanosome distribution is shown in A. Error bars indicate means ± SE of three independent experiments (*n* > 25 cells for each experiment), which were performed on different days. ***P < 0.001 (one-way analysis of variance and Tukey’s test). **(C)** Expression of RAB27A proteins in melan-ash cells is shown in A as determined by immunoblotting with the antibodies indicated. The positions of the molecular mass markers (in kilodaltons) are shown on the left. Source data are available for this figure: SourceData F4.

To further determine whether novel RAB27A(Ser115Arg) and previously reported RAB27A(Ser106PhefsTer18) variants (19) can support melanosome transport in melanocytes, we transiently expressed these RAB27A variants with EGFP-tag in RAB27A-deficient melan-ash cells (18), where melanosomes were aggregated in the nuclear region (Fig. 4 A, top row). The results showed that the RAB27A(Ser115Arg) variant completely restored the peripheral melanosome distribution, similar to RAB27A(WT) (Fig. 4 A, insets in the second and third rows; Fig. 4 B), consistent with the fact that patient 2 did not exhibit any pigmentation defects. However, the RAB27A(Ser115Arg) variant appeared to be less localized to melanosomes than RAB27A(WT) (Fig. 4 A, insets in the second and third rows). In contrast, the RAB27A(Ser106PhefsTer18) variant was hardly expressed in melan-ash cells, and we detected only a few EGFP-positive cells, all of which showed a perinuclear aggregation phenotype (Fig. 4 A, bottom row). Actually, we did not detect a RAB27A(Ser106PhefsTer18) band on immunoblot analysis (Fig. 4 C). Thus, a truncated form of RAB27A by the frameshift variant was likely not stably expressed in melanocytes.

### Effect of the Ser115Arg variant of RAB27A on binding activity toward RAB27A effectors

**Figure 5.**
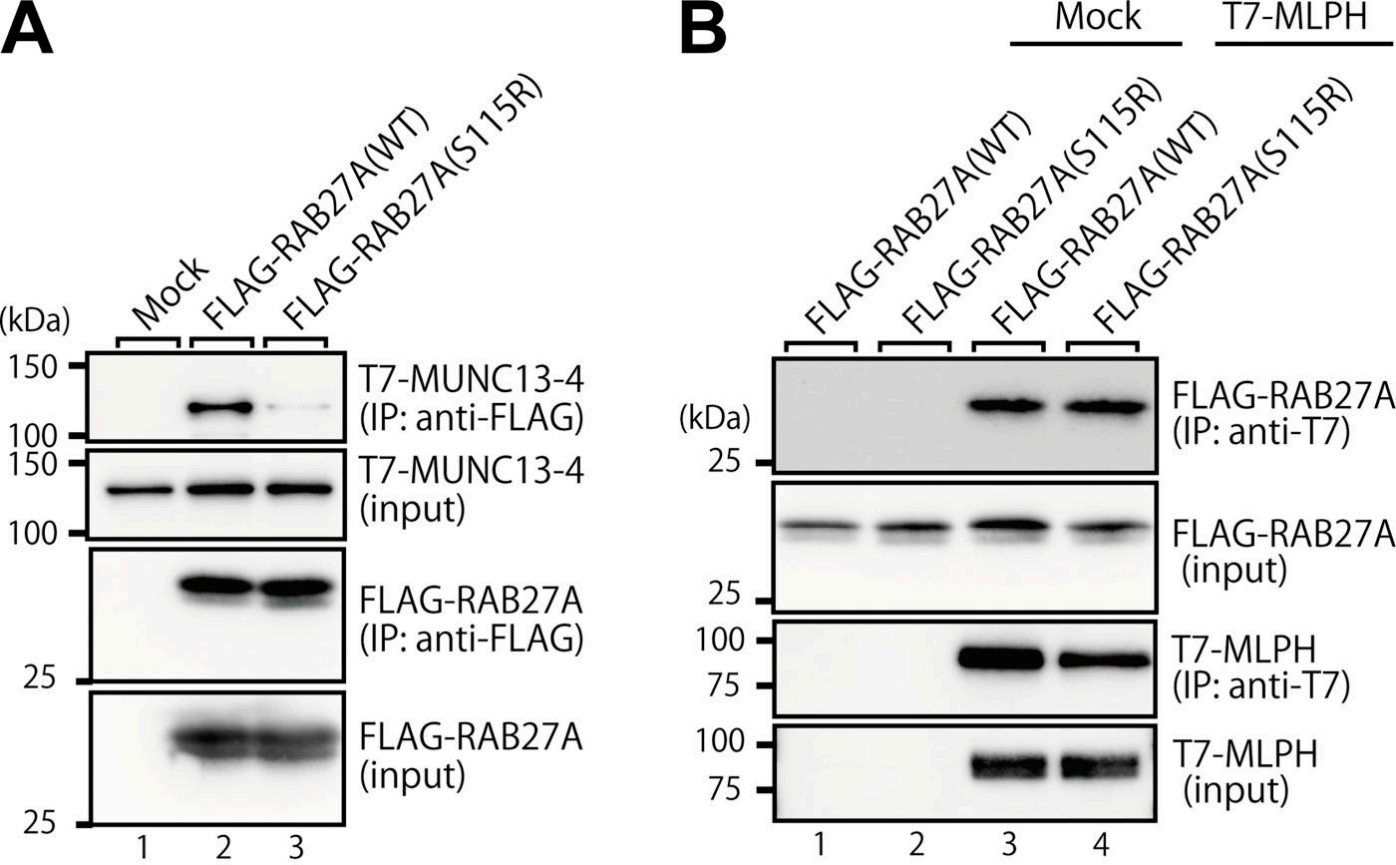
**RAB27A effector-binding activities of RAB27A(Ser115Arg)**. **(A)** Interaction between T7-MUNC13-4 and FLAG-RAB27A(WT or Ser115Arg; indicated as S115R). **(B)** T7-MLPH and FLAG-RAB27A(WT or Ser115Arg). These protein interactions in COS-7 cell lysates were analyzed by co-immunoprecipitation assays using anti-FLAG tag antibody-conjugated agarose beads (A) or anti-T7 tag antibody-conjugated agarose beads (B). Co-immunoprecipitated T7-MUNC13-4 (or FLAG-RAB27A) and immunoprecipitated (IP) FLAG-RAB27A (or T7-MLPH) were detected by immunoblotting with HRP-conjugated anti-T7 tag antibody and anti-FLAG tag antibody (or anti-FLAG tag antibody and anti-T7 tag antibody), respectively. The positions of the molecular mass markers (in kilodaltons) are shown on the left. Data in A and B are representative of the data obtained in three independent experiments, and similar results were obtained in each experiment. Source data are available for this figure: SourceData F5.

To evaluate the effect of the Ser115Arg variant of RAB27A on its effector-binding ability, we performed co-immunoprecipitation assays by expressing FLAG-tagged RAB27A and T7-tagged RAB27A effectors in COS-7 cells. The results showed that the Ser115Arg variant of RAB27A clearly reduced the binding activity toward MUNC13-4 (Fig. 5 A), which perfectly matches the hemophagocytic phenotype of patient 2. In contrast, however, it did not affect the binding activity toward MLPH (Fig. 5 B), consistent with the fact that RAB27A(Ser115Arg) fully supports actin-based melanosome transport and maintains peripheral melanosome distribution in melan-ash cells (Fig. 4). These results taken together indicated that the Ser115Arg variant of RAB27A specifically reduces its effector-binding activity toward MUNC13-4 (RAB27A effector in CTLs [9, 10, 11]), but not toward MLPH (RAB27A effectors in melanocytes [4, 5, 6, 20]).

## Discussion

This study shows that certain combinations of *RAB27A* alleles can separate pigmentation from cytotoxic lymphocyte function. The result is GS2 without hypopigmentation but with a high risk of HLH. Functional data support an allele-combination mechanism: Trp73Arg and Ser106PhefsTer18 behaved as a loss-of-function for both MLPH and MUNC13-4 binding. Ser115Arg and Val143Ala preserved MLPH binding but reduced MUNC13-4 interaction. In melanocytes, this maintained peripheral melanosome transport, whereas in cytotoxic lymphocytes, it impaired granule priming and release (Figs. 2, 3, 4, and 5) (4, 5, 6, 7, 8, 9, 10, 11). Hair-shaft TEM in patient 1 confirmed preserved melanosome distribution, supporting this model (Fig. 1 B) (12, 13, 14, 15).

These findings extend known genotype–phenotype correlations in GS2. Variants at or near Trp73 have been linked to defective effector engagement, and Trp73 variants show diffuse localization and loss of effector binding in melanocytes (21). Val143Ala, located outside canonical nucleotide-binding motifs, selectively impairs MUNC13-4 binding but spares MLPH. This explains preserved pigmentation despite defective immunity (11). Large cohort analysis has shown that missense *RAB27A* variants often act as partial loss-of-function alleles, leading to GS2 with preserved pigmentation (sine albinism) while still predisposing to severe HLH (3). Together, these data support a residue-level, tissue-specific model: alleles that preserve MLPH engagement allow melanosome transport, whereas alleles that reduce MUNC13-4 interaction abrogate cytotoxic granule exocytosis (8, 9, 10, 11).

**Figure S1.**
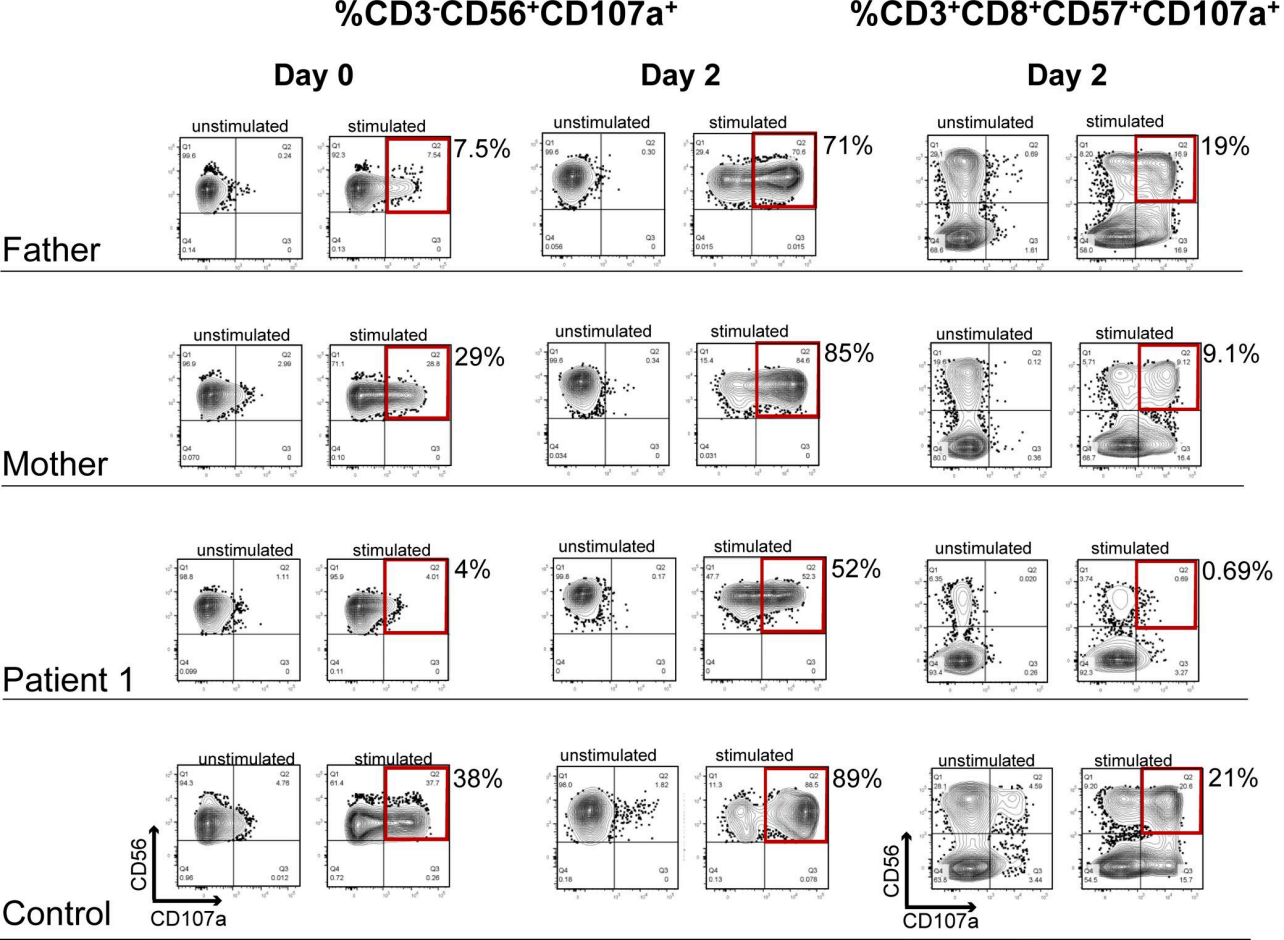
**NK and CD8** ^**+**^ **T cell degranulation assays in patient 1 and family members.** Representative flow cytometric plots from patient 1, a healthy control, and the patient’s parents. PBMCs were analyzed either at day 0 without prior cytokine stimulation or after 48 h of culture with IL-2 (day 2). Degranulation was assessed after 2 h under unstimulated conditions or following target-cell stimulation. Left and middle panels show NK cell degranulation, quantified as CD3^−^CD56^+^CD107a^+^ cells. NK cell degranulation was assessed at day 0 (without IL-2) and at day 2 (after 48 h of IL-2 culture), under unstimulated conditions or after co-culture with K562 target cells. Right panels show CD8^+^ cytotoxic T cell degranulation, quantified as CD3^+^CD8^+^CD57^+^CD107a^+^ cells, assessed at day 2 after IL-2 culture, under unstimulated conditions or after co-culture with P815 cells alone or with P815 cells in the presence of anti-CD3 (OKT3). Percentages of CD107a-positive cells are indicated within the gated regions. Markedly reduced degranulation responses were observed only in the patient, whereas normal degranulation was preserved in the healthy control and both parents, including the father carrying p.Gly94Ser and p.Trp73Arg in cis. Experiments were performed once for each individual due to clinical constraints. The patient 1 and healthy control plots on day 0 shown here are reproduced from Fig. 1 for direct comparison with the parental samples.

In silico pathogenicity prediction and population frequency data for all identified *RAB27A* variants are summarized in Table S1. Although structural and in silico approaches have been used to infer RAB27A–effector interactions (22, 23), such analyses were not performed for the individual variants in this study. In patient 1, two rare missense variants (p.Gly94Ser and p.Trp73Arg) were present in cis. Despite damaging in silico predictions, p.Gly94Ser is classified as a variant of uncertain significance, occurs at very low population frequency, and was observed in an unaffected carrier (Table S1). Consistently, CD3^−^CD56^+^NK and CD3^+^CD8^+^T cell degranulation assays in the father were normal (Fig. S1), supporting low clinical impact of the paternal allele in the heterozygous state and haplosufficiency of *RAB27A*. Although we cannot exclude the possibility that two predicted damaging variants on the same allele reduce overall RAB27A function, such an effect appears insufficient to cause disease in isolation. Accordingly, our data are most consistent with partial loss-of-function rather than dominant-negative effects in GS2.

Importantly, partial loss-of-function missense variants are increasingly recognized in GS2 and account for many sine albinism cases (1, 2, 3). Population-level data support the frequency of this phenotype. In a cohort of 149 patients with *RAB27A* deficiency, hypopigmentation was absent in a substantial minority, and CNS disease occurred in nearly half of the patients, highlighting that pigmentation does not predict immune severity (3). Clinically, visible pigmentation does not exclude *RAB27A* deficiency. In children with fulminant or recurrent HLH and very low CD107a responses, early *RAB27A* testing and functional annotation should guide timely definitive therapy (1, 2, 3, 12, 13, 14, 15).

CNS disease in GS2 is often associated with systemic immune dysregulation in HLH, but may not always be fully explained by secondary neuroinflammation alone. CNS involvement is well documented in pediatric GS2 and HLH, with neurological features such as white-matter lesions, seizures, and focal deficits frequently described (1, 2, 3). In our series, a JAK inhibitor (ruxolitinib) was associated with defervescence and transient neurological stabilization in one patient; however, its independent contribution cannot be determined because it was administered in combination with immunochemotherapy. Pediatric HLH studies report fever control and steroid/etoposide-sparing effects with ruxolitinib, but evidence is still limited, and its role in CNS-HLH has not been established (24, 25). However, current evidence remains limited to small case series, and the specific role in CNS-HLH is not yet established (16). Additional cases collected through multicenter registries and prospective standardized protocols are needed.

This study had several limitations. First, we described only two patients. Functional assays relied on tagged overexpression systems in murine melanocytes, not on patient-derived melanocytes or cytotoxic lymphocytes. Additionally, hair-shaft TEM was performed in only one patient. Nevertheless, these cases add to evidence that compound-heterozygous *RAB27A* missense variants can produce GS2 sine albinism, underscoring the importance of genetic and functional testing particularly when pigmentation is normal.

In conclusion, compound-heterozygous *RAB27A* variants can impair cytotoxic granule release while preserving melanosome transport, resulting in GS2 with sine albinism and a high risk of HLH. The novel missense variants identified here broaden the spectrum of pathogenic *RAB27A* alleles. An integrated approach that combines genetic testing with standardized functional assays improves variant interpretation and supports timely decisions on definitive treatment, even in patients with sine albinism.

## Materials and methods

### Study approval and consent

This study was approved by the Institutional Review Boards of Hirosaki University and Kyoto University. Written informed consent was obtained from parents or legal guardians for clinical data use and research assays, in accordance with the Declaration of Helsinki.

### Patients and clinical evaluations

Genetic analysis of the genes responsible for familial HLH, including *PRF1*, *UNC13D*, *STX11*, *STXBP2*, *FAAP24*, *SLC7A7*, *LYST*, *RAB27A*, *AP3B1*, *AP3D1*, *SH2D1A*, and *XIAP*, was performed using a targeted HLH panel (Kazusa DNA Research Institute, Kisarazu, Japan) as described previously (26).

### Degranulation assay

To quantify granule exocytosis by NK cells, 2 × 10^5^ peripheral blood mononuclear cells (PBMCs) that were freshly isolated or stimulated with IL-2 (100 U/ml) for 36–48 h were co-cultured with or without 2 × 10^5^ K562 cells and incubated in complete medium (RPMI 1640 medium supplemented with 2 mM L-glutamine and 10% fetal calf serum) for 2 h at 37°C in 5% CO_2_. For CTL degranulation analyses, 2 × 10^5^ PBMCs stimulated with IL-2 (100 U/ml) for 36–48 h were cultured with 2 × 10^5^ P815 cells with or without 0.5 μg/ml anti-CD3 mAb (OKT3). The cells were resuspended in phosphate-buffered saline supplemented with 0.5% bovine serum albumin and 2 mM ethylene diamine tetra-acetic acid (EDTA); stained with anti-CD3, anti-CD8, anti-CD16, anti-CD56, anti-CD57, and anti-CD107a monoclonal antibodies; and then analyzed by flow cytometry. Surface CD107a expression on CD3^−^CD56^+^ NK cells and CD3^+^CD8^+^CD57^+^ T cells was quantified as an indicator of lysosomal degranulation (27).

### Electron microscopy

Specimens were fixed with a mixture of 2.5% glutaraldehyde and 2% paraformaldehyde in 0.1 M phosphate buffer (PB) for several days at 4°C. After rinsing with 0.1 M PB, the specimens were post-fixed with 1% OsO_4_ for 2 h on ice and then dehydrated via an alcohol series. After treatment with propylene oxide for resin infiltration, the specimens were embedded in epoxy resin at 60°C for 3 days. Semithin sections sliced at 500 nm were stained with toluidine blue. Ultrathin sections were sliced at 70 nm and mounted on the copper sheet meshes (single-hole; φ1.2, Nisshin EM Co., Ltd.) coated with the formvar film. The sections were then stained with uranyl acetate and lead citrate. Observations were performed using a transmission electron microscope (JEM-1400; JEOL Ltd.) and a MultiScan BioScan CCD Camera (Model 792, Gatan, Inc.).

### Reagents and antibodies

Anti-FLAG tag mouse monoclonal (M2) antibody-conjugated agarose beads (A2220; Sigma-Aldrich), horseradish peroxidase (HRP)-conjugated anti-FLAG tag mouse monoclonal (M2) antibody (A8592; Sigma-Aldrich), HRP-conjugated anti-T7 tag mouse monoclonal antibody (69048; Novagen, Merck KGaA), anti-T7 tag antibody-conjugated agarose (Novagen, Merck KGaA), and HRP-conjugated anti-GFP rabbit polyclonal antibody (598-7; MBL) were obtained commercially.

### Plasmid construction

The cDNA encoding mouse RAB27A(Trp73Arg) was prepared by standard molecular biology techniques using the following mutagenic oligonucleotides (substituted nucleotides in bold): 5′-CAC​CTG​CAG​TTA**A**GGG​ACA​CGG​CGG​GGC​AG-3′ (sense) and 5′-CTG​CCC​CGC​CGT​GTC​CC**T**TAA​CTG​CAG​GTG-3′ (antisense). The RAB27A(Trp73Arg) cDNA was subcloned into the pEF-FLAG tag expression vector (28) and the pEGFP-C1 vector (Takara Bio Inc., Shiga, Japan). The RAB27A(Trp73Arg/Gln78Leu/Cys219Ala/Cys221Ala) cDNA was similarly prepared and subcloned into the pGBD-C1 vector (29). Other expression plasmids, including pAct2-MLPH-SHD (Slp homology domain; amino acids 1–153), pEF-T7-MLPH, pEF-T7-MUNC13-4, and pEF-FLAG-RAB27A, were prepared as described previously (30, 31, 32). The cDNAs encoding human RAB27A(Ser115Arg) and RAB27A(Ser106PhefsTer18) were also prepared using the standard molecular biology techniques, with human RAB27A cDNA (33) as a template and the following mutagenic oligonucleotides (substituted nucleotides in bold): 5′-AGA​AAC​TGG​ATA​AG**G**CAG​CTA​CAG​ATG​CAT-3′ (Ser115Arg, sense), 5′-ATG​CAT​CTG​TAG​CTG**C**CTT​ATC​CAG​TTT​CT-3′ (Ser115Arg, antisense), 5′-GAT​CTG​ACA​AAT​GAG​CAG​TTT​CCT​CAA​TGT​CAG-3′ (Ser106PhefsTer18, sense), and 5′-CTG​ACA​TTG​AGG​AAA​CTG​CTC​ATT​TGT​CAG​ATC-3′ (Ser106PhefsTer18, antisense). The WT and variant human RAB27A cDNAs were subcloned into the pEF-FLAG tag expression vector (28) and the pEGFP-C1 vector. All these plasmids were confirmed by DNA sequencing.

### Cell cultures and transfection

The RAB27A-deficient *ashen* mouse-derived immortal melanocyte cell line (named melan-ash) was obtained from the Wellcome Trust Functional Genomics Cell Bank at St George’s, University of London, and cultured as described previously (18). COS-7 cells were cultured at 37°C in Dulbecco’s modified Eagle’s medium supplemented with 10% fetal bovine serum, 100 U/ml penicillin G, and 100 μg/ml streptomycin in a 5% CO_2_ incubator. Cells were transfected with plasmid DNAs using Lipofectamine 2000 or 3000 (Thermo Fisher Scientific) according to the manufacturer’s instructions.

### Yeast two-hybrid assays

The yeast strain, medium, culture conditions, and transformation protocol used were as described previously (29). Yeast two-hybrid assays were performed using pGBD-C1-RAB27A (WT or Trp73Arg) carrying Gln78Leu/Cys219Ala/Cys221Ala variants or pGBD-C1-RAB38(Gln69Leu)ΔCys and pAct2-MLPH-SHD as described previously (30, 34). Constitutively active (Gln/Leu) and geranylgeranylation-deficient (Cys/Ala or ΔCys) mutants of RAB27A and RAB38 were used for yeast two-hybrid assays to promote efficient RAB–effector interactions. Yeast cells on a selection medium (SC-AHLW: synthetic complete [SC] medium lacking adenine, histidine, leucine, and tryptophan) and a growth medium (SC-LW) were incubated at 30°C for 4 and 2 days, respectively.

### Immunofluorescence and melanosome distribution assays

Precisely 2 days after transfecting pEGFP-C1 plasmids into melan-ash cells, the cells were fixed with 4% paraformaldehyde for 10 min, permeabilized with 0.05% saponin for 30 min, blocked with 1% bovine serum albumin for 30 min, and stained with DAPI (1 µg/ml) for 1 h. Fluorescence images and the corresponding bright-field images were captured at random with an FV1000D confocal fluorescence microscope and Fluoview software (Evident/Olympus,). The percentage of cells showing peripheral melanosome distribution was calculated after a manual cell count. Cells in which >50% of the melanosomes were present around the nucleus were judged to be “aggregated” (i.e., typical phenotype in melan-ash cells), and the rest of the cells were judged to be “dispersed” (i.e., normal phenotype in WT cells) as described previously (20).

### Co-immunoprecipitation assays in COS-7 cells

COS-7 cells were transfected with pEF-FLAG-RAB27A (WT or Ser115Arg) or pEF-T7-MLPH using Lipofectamine 2000. A day after transfection, the cells were lysed with a lysis buffer (50 mM HEPES-KOH, pH 7.2, 150 mM NaCl, 1 mM MgCl_2_, and 1% Triton X-100 supplemented with cOmplete EDTA-free protease inhibitor mixture [Roche]). The lysates of MLPH-expressing cells were incubated for 1 h at 4°C with anti-T7 tag antibody-conjugated agarose beads. After washing three times with a washing buffer (50 mM HEPES-KOH, pH 7.2, 150 mM NaCl, 1 mM MgCl_2_, and 0.1% Triton X-100), the beads were incubated for 1 h at 4°C with the lysates of RAB27A (WT or Ser115Arg)-expressing cells. After washing the beads three times with the washing buffer again, proteins bound to the beads were analyzed by immunoblotting as described previously (13). Immunoreactive bands were visualized by enhanced chemiluminescence, and images were captured by a ChemiDoc Touch Imaging System (Bio-Rad). Interactions between MUNC13-4 and RAB27A were also evaluated by co-immunoprecipitation assays as described previously (14).

### Statistical analysis

Statistical analysis was performed using one-way analysis of variance followed by Tukey’s test. P < 0.05 was considered statistically significant (***, P < 0.001). NS, not significant (P > 0.05).

### Online supplemental material

Supplemental material for this article includes Fig. S1 and Table S1. Fig. S1 shows NK and CD8^+^ T cell degranulation assays in patient 1 and family members. Table S1 shows genetic and in silico characterization of RAB27A variants identified in this study.

## Ethics approval

This study was performed in accordance with the Declaration of Helsinki and approved by the Institutional Review Boards of Hirosaki University Graduate School of Medicine (No. 2022-1033-4).

## Informed consent statement

Written informed consent was obtained from the parents or legal guardians of all participants.

## Supplementary Material

Table S1 shows genetic and in silico characterization of RAB27A variants identified in this study.

SourceData F3 is the source file for Fig. 3.

SourceData F4 is the source file for Fig. 4.

SourceData F5 is the source file for Fig. 5.

## Data Availability

All data supporting the findings of this study are available within the article. Individual-level clinical and genetic data are not publicly available due to ethical and privacy considerations but are available from the corresponding author upon reasonable request. All *RAB27A* variants identified in this study have been submitted to ClinVar (accession numbers SCV007538185, SCV007538186, SCV007538187, and SCV007538188).
