# Supplementary figures and images for: Functional characterization of Griscelli syndrome type 2 sine albinism in Japanese patients
Source: J Hum Immun. 2026 May 7;2(4):e20250270. doi: 10.70962/jhi.20250270 (PMC13177389; doi:10.70962/jhi.20250270)

Fig. 3B - top panel

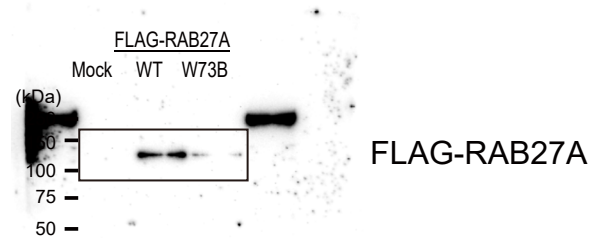

Fig. 3B - second panel

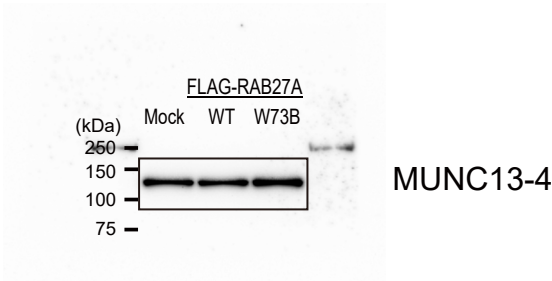

Fig. 3B - third panel

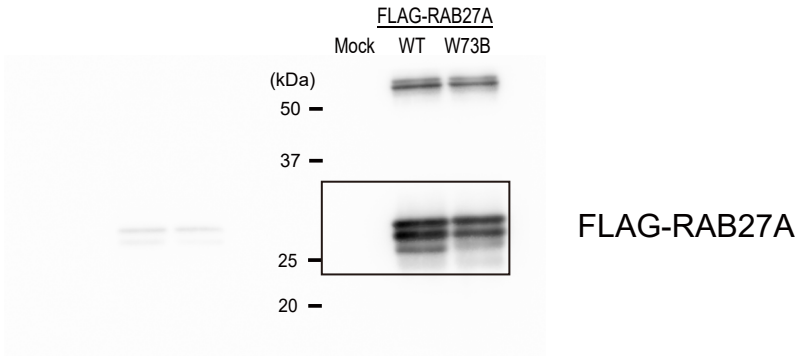

Fig. 3B - bottom panel

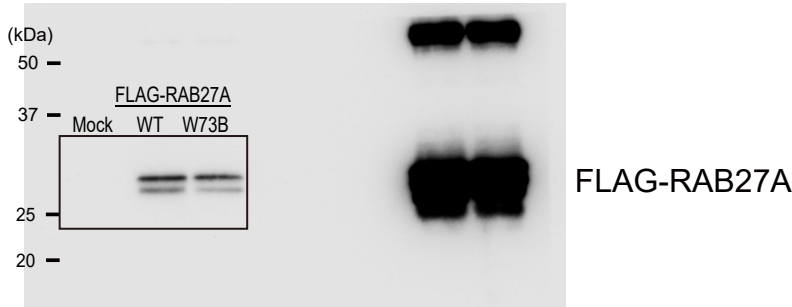

Supplement: SourceData F3 — is the source file for Fig. 3. [file jhi_20250270_sourcedataf3.pdf]

Fig. 4C - top panel

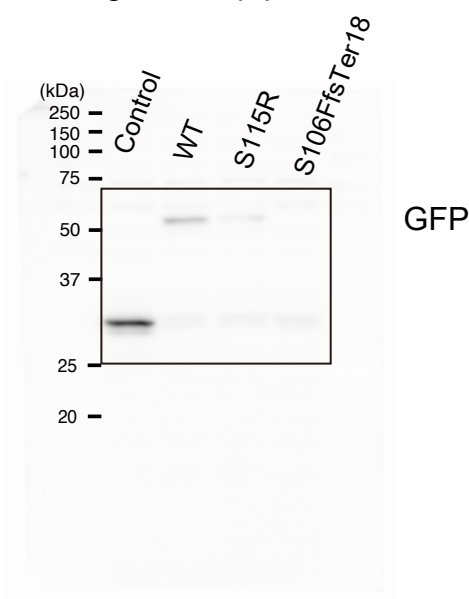

Fig. 4C - bottom panel

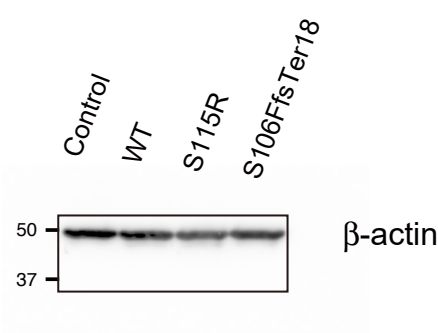

Source data for Figure 4C

Supplement: SourceData F4 — is the source file for Fig. 4. [file jhi_20250270_sourcedataf4.pdf]
