# Supplementary material for: Functional characterization of Griscelli syndrome type 2 sine albinism in Japanese patients
Source: J Hum Immun. 2026 May 7;2(4):e20250270. doi: 10.70962/jhi.20250270 (PMC13177389; doi:10.70962/jhi.20250270)
Supplement: SourceData F5 — is the source file for Fig. 5. [file jhi_20250270_sourcedataf5.pdf]

Source Data F5

Fig. 5A - top panel

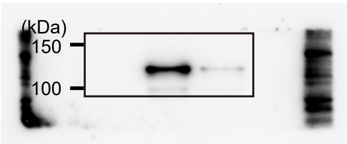

Fig. 5A - second panel

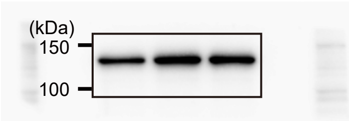

Fig. 5A - third panel

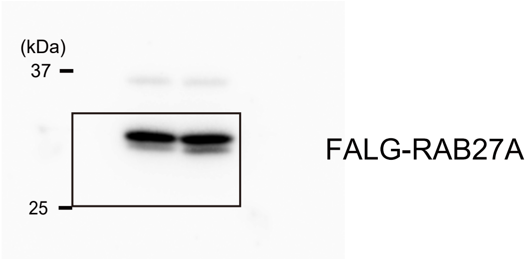

Fig. 5A - bottom panel

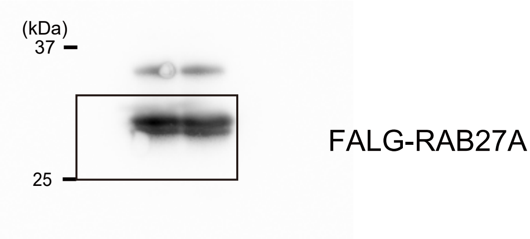

Fig. 5B - top panel

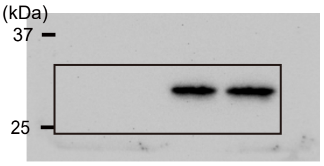

Fig. 5B - second panel

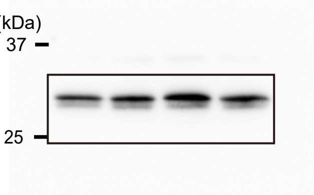

Fig. 5B - third panel

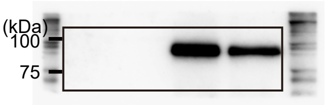

Fig. 5B - bottom panel

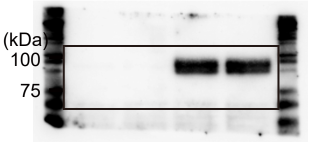

Source data for Figure 5AB
